# Incidence and Outcomes of Acute Respiratory Distress Syndrome in Brain-Injured Patients Receiving Invasive Ventilation: A Secondary Analysis of the ENIO Study

**DOI:** 10.1177/08850666231194532

**Published:** 2023-08-11

**Authors:** Shaurya Taran, Robert D. Stevens, Bastien Perrot, Victoria A. McCredie, Raphael Cinotti, Karim Asehnoune, Paolo Pelosi, Chiara Robba, for the ENIO Study Group, on behalf of the PROtective VENTilation network, the European Society of Intensive Care Medicine, the Colegio Mexicano de Medicina Critica, the Atlanréa group, and the La Société Française d’Anesthésie et de Réanimation (SFAR) research network

**Affiliations:** 1 Interdepartmental Division of Critical Care Medicine, 7938 University of Toronto, Toronto, ON, Canada; 2 Department of Neurology, Massachusetts General Hospital, Harvard Medical School, Boston, MA, USA; 3 Department of Anesthesiology and Critical Care Medicine, 1500 Johns Hopkins University School of Medicine, Baltimore, MD, USA; 4 Department of Neurology, 1500 Johns Hopkins University School of Medicine, Baltimore, MD, USA; 5 Department of Biomedical Engineering, Whiting School of Engineering, Johns Hopkins University, Baltimore, MD, USA; 6 UMR 1246 MethodS in Patient-centered outcomes and HEalth REsearch, SPHERE, 27045 Nantes Université, Tours Université, Nantes, France; 7 Department of Anaesthesia and Critical Care, CHU Nantes, 27045 Nantes Université, Hôtel-Dieu, Nantes, France; 8 Anesthesia and Critical Care, San Martino Policlinico Hospital, IRCCS for Oncology and Neuroscience, Genoa, Italy; 9 Department of Surgical Sciences and Integrated Diagnostics, University of Genoa, Genoa, Italy

**Keywords:** acute respiratory distress syndrome, mechanical ventilation, traumatic brain injury, subarachnoid hemorrhage, intracranial hemorrhage

## Abstract

**Background:** Acute respiratory distress syndrome (ARDS) is an important pulmonary complication in brain-injured patients receiving invasive mechanical ventilation (IMV). We aimed to evaluate the incidence and association between ARDS and clinical outcomes in patients with different forms of acute brain injury requiring IMV in the intensive care unit (ICU). **Methods:** This was a preplanned secondary analysis of a prospective, multicenter, international cohort study (NCT 03400904). We included brain-injured patients receiving IMV for ≥ 24 h. ARDS was the main exposure of interest and was identified during index ICU admission using the Berlin definition. We examined the incidence and adjusted association of ARDS with ICU mortality, ICU length of stay, duration of IMV, and extubation failure. Outcomes were evaluated using mixed-effect logistic regression and cause-specific Cox proportional hazards models. **Results:** 1492 patients from 67 hospitals and 16 countries were included in the analysis, of whom 137 individuals developed ARDS (9.2% of overall cohort). Across countries, the median ARDS incidence was 5.1% (interquartile range [IQR] 0-10; range 0-27.3). ARDS was associated with increased ICU mortality (adjusted odds ratio (OR) 2.66; 95% confidence interval [CI], 1.29-5.48), longer ICU length of stay (adjusted hazard ratio [HR] 0.59; 95% CI, 0.48-0.73), and longer duration of IMV (adjusted HR 0.54; 95% CI, 0.44-0.67). The association between ARDS and extubation failure approached statistical significance (adjusted HR 1.48; 95% CI 0.99-2.21). Higher ARDS severity was associated with incrementally longer ICU length of stay and longer cumulative duration of IMV. Findings remained robust in a sensitivity analysis evaluating the magnitude of unmeasured confounding. **Conclusions:** In this cohort of acutely brain-injured patients, the incidence of ARDS was similar to that reported in other mixed cohorts of critically ill patients. Development of ARDS was associated with worse outcomes.

## Introduction

Many patients with acute brain injury (ABI) require invasive mechanical ventilation (IMV) and admission to an intensive care unit (ICU).^1,2^ Non-neurologic complications, including the acute respiratory distress syndrome (ARDS),^3–6^ are an important source of additional morbidity and mortality in this population. ARDS occurs in 20% to 30% of patients with ABI,^7–9^ whereas its incidence is 10% in mixed cohorts of critically ill patients admitted to the ICU.^10^ Brain injury is thought to mediate susceptibility to ARDS through immune suppression, sympathetic activation, and release of pro-inflammatory cytokines following the initial neurologic insult.^11–13^ In patients receiving IMV, ventilator-induced lung injury (VILI) may further contribute to ARDS risk; this remains a concern in brain-injured patients, who have been shown to receive higher tidal volumes and higher plateau pressures compared to patients without ABI.^14^

To date, few studies have evaluated the incidence, impact, and association of ARDS with clinical outcomes in brain-injured patients.^15–18^ A recent systematic review found that current data on ARDS incidence, morbidity, and mortality following ABI are largely derived from single-center cohorts of patients with traumatic brain injury (TBI) and may be driven disproportionately by patients who die before liberation from IMV.^19^ The existing literature also does not make clear whether (and to what degree) outcomes are modified by regional effects, such as country of management.

We therefore conducted a secondary analysis of a prospective, multicenter, international cohort study performed in patients with ABI surviving beyond liberation from IMV.^20^ We sought to describe the incidence of ARDS in this population, report regional variation in ARDS incidence, and evaluate the association of ARDS with clinical outcomes after accounting for regional effects. We hypothesized that ARDS would be associated with excess morbidity and mortality in this population.

## Materials and Methods

### Data Sources

This was a preplanned secondary analysis of Extubation strategies in Neuro-Intensive care unit patients and association with Outcomes (ENIO).^20^ ENIO was a multicenter prospective observational study evaluating extubation outcomes in 1512 acutely brain-injured patients receiving IMV across 73 ICUs worldwide. The study was conducted from June 2018 to November 2020. Local ethics committees approved ENIO at all participating centers following central ethics approval in November 2017 (Groupe Nantais d’Ethique dans le Domaine de la Santé, IRB No. 7-11-2017). Informed consent or waivers of consent were obtained as per local regulations, and the study was conducted in accordance with the Declaration of Helsinki. This sub-study proposal was approved by the ENIO steering committee; no further ethical approval was deemed necessary. Results are presented according to the Strengthening of Reporting of Observational Studies in Epidemiology (STROBE) statement.^21^

### Study Population and Data Collection

Full details of patient inclusion and exclusion criteria are provided in the original report.^20^ Briefly, patients with ABI (defined as patients with aneurysmal subarachnoid hemorrhage [aSAH], TBI, intracranial hemorrhage [ICH], acute ischemic stroke, brain tumor, or central nervous system infection), aged ≥18 years, who received IMV for ≥24 h in an ICU, and in whom ventilator liberation (defined as extubation trial or primary tracheostomy) was attempted, were included in ENIO. Patients were excluded if they were admitted with major chest trauma (defined as an abbreviated injury scale score ≥3), cardiac arrest, motor neuron disease, muscular dystrophy, myasthenia gravis, Guillain-Barré syndrome, or spinal cord injury above T4; were pregnant; had extubation performed at the end of life; had chronic home oxygen use or stage III/IV chronic obstructive pulmonary disease per the Global Initiative for Obstructive Lung Disease (GOLD) criteria; had a tracheostomy present before ICU admission; or had withdrawal of life-sustaining treatments in the first 24 h after ICU admission. Patients who died before attempted liberation from IMV were also excluded from the original study.^20^

All patients from ENIO with non-missing information on ARDS status were included in this analysis. Data in the original report were collected at ICU admission, during ICU stay for the index admission, at ICU discharge, and at hospital discharge. Available data included pre-existing medical comorbidities, neurologic diagnosis on admission, ICU events, sedation strategy, ventilatory parameters and arterial blood gases (each measured on days 1, 3, and 7 of IMV), and data on extubation readiness. All patients were followed until death or hospital discharge, with no dropouts.

### Definition of ARDS

ARDS was defined as bilateral pulmonary opacities and hypoxemic respiratory failure developing within 7 days of a recognized clinical insult, and not attributable to fluid overload or cardiogenic pulmonary edema, as per the Berlin definition.^22^ Patients with ARDS were identified by study personnel from each hospital site following review of clinical data and chest radiographs. ARDS severity was further classified as mild, moderate, or severe, according to the partial pressure of oxygen to fraction of inspired oxygen concentration (PaO_2_/FiO_2_) ratio (mild ARDS, 200 < PaO_2_/FiO_2_ ≤ 300; moderate ARDS, 100 < PaO_2_/FiO_2_ ≤ 200; severe ARDS, PaO_2_/FiO_2_ ≤ 100).^22^ Severity was designated using the worst PaO_2_/FiO_2_ ratio after the patient met Berlin definition criteria for ARDS.

### Objectives

The co-primary aims of this study were to identify the incidence of ARDS in patients with ABI and examine variation in ARDS incidence by world region. The secondary aim was to report the association between ARDS and clinical outcomes.

### Clinical Outcomes

The primary clinical outcome was ICU mortality, defined as death at any timepoint from any cause during ICU admission. Additional outcomes were ICU length of stay (LOS), duration of IMV, and extubation failure. Duration of IMV was recorded as the overall number of days spent receiving mechanical ventilation, including ventilatory days arising after subsequent reintubations. Extubation failure was defined as the unplanned need for reintubation at any timepoint in the ICU following the index extubation attempt. All study outcomes were evaluated for the index ICU admission.

### Statistical Analyses

Descriptive statistics were used to examine characteristics of the patient sample. Data are summarized as median (interquartile range) for continuous data and count (%) for categorical data. Data were compared using the Wilcoxon rank-sum test, chi-square test, or Fisher's exact test as appropriate.

*Model Development*. Clinical outcomes were evaluated using mixed models with patient-level covariates and random effects for country. All models included ARDS as the primary exposure of interest and were adjusted using patients’ baseline characteristics, medical comorbidities, neurologic admission diagnosis, and clinical course in the ICU. Patient covariates were selected based on subject matter knowledge, literature review, and related research.^23–25^ Putative associations between ARDS and outcomes were defined using a directed acyclic graph (eFigure 1). The final pool of adjustment predictors for each model reflected a balance between clinical relevance and percent missingness.

ICU mortality was evaluated using a mixed-effect logistic regression model. ICU LOS, duration of IMV, and extubation failure were reported using mixed-effect, cause specific Cox proportional hazards models. To allow for expected variation across clusters, Cox models included a shared frailty term for patients managed in the same country. The proportional hazards assumption was tested by plotting the scaled Schoenfeld residuals against analysis time. Competing risks in time-to-event analyses were handled via censoring. For assessment of model fit, mixed models were compared to corresponding conventional regression models (which excluded the random intercept term) using the likelihood ratio test.^26^ All models were fitted using complete case analysis without additional procedures to account for missing data, given that <5% of data on covariates and outcomes of interest was missing.^27,28^ Associations were reported using adjusted odds ratios (OR) or hazard ratios (HR) with corresponding 95% confidence intervals (CI), as appropriate. Additional methods and sample codes are provided in eAppendices 1 to 2.

*Additional Analyses*. To evaluate the impact of ARDS severity on clinical outcomes, we refitted models with ARDS defined as mild, moderate, or severe, using the worst PaO_2_/FiO_2_ ratio recorded during ICU admission. We also examined the association between ARDS and ICU mortality (the primary clinical outcome) in subgroups defined by neurologic diagnosis at ICU admission. Four diagnoses were examined: TBI, aSAH, ICH, and “Others” (defined as patients with any of the following: acute ischemic stroke, brain tumor, brain abscess, empyema, or encephalitis). Subgroup models were fitted using fewer covariates to prevent overfitting. All associations were reported against the reference category of patients without ARDS.

In a sensitivity analysis, we calculated *E*-values to evaluate the impact of unmeasured confounders on the association between ARDS and clinical outcomes. The *E*-value is defined as the minimum strength of association that an unmeasured confounder should have with both the exposure and outcome of interest to explain away the estimated association.^29^ We reported the *E*-value together with the lower bound of the 95% confidence interval.

All reported *P* values were two-sided with a threshold of <.05 for statistical significance. Analyses were performed using STATA v.18.0 (StataCorp) and R v.4.0.3 (R Foundation for Statistical Computing).

## Results

### Cohort Characteristics

**Figure 1.**
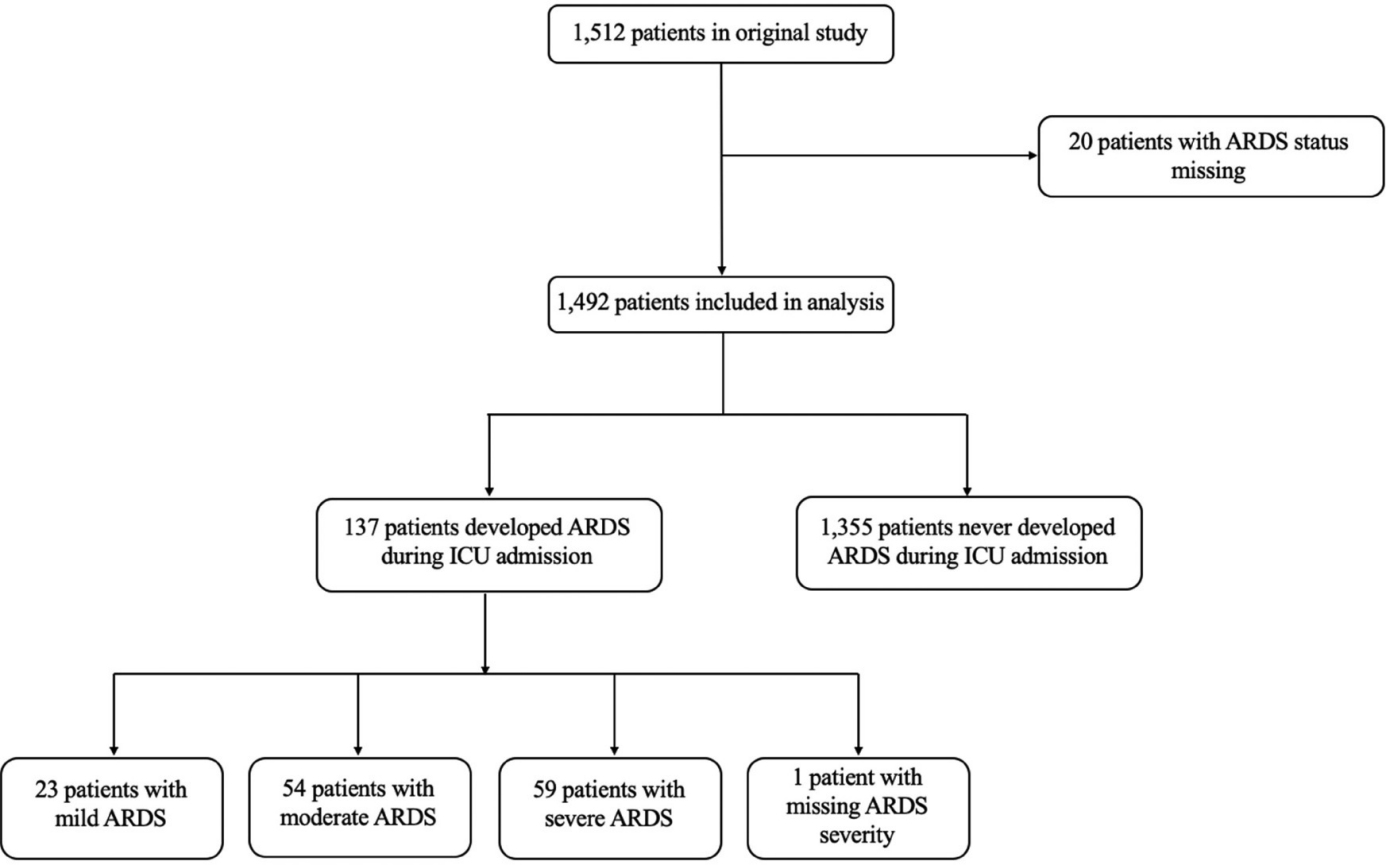
Study flow diagram.

Abbreviations: ARDS, acute respiratory distress syndrome; ICU, intensive care unit.

**Figure 2.**
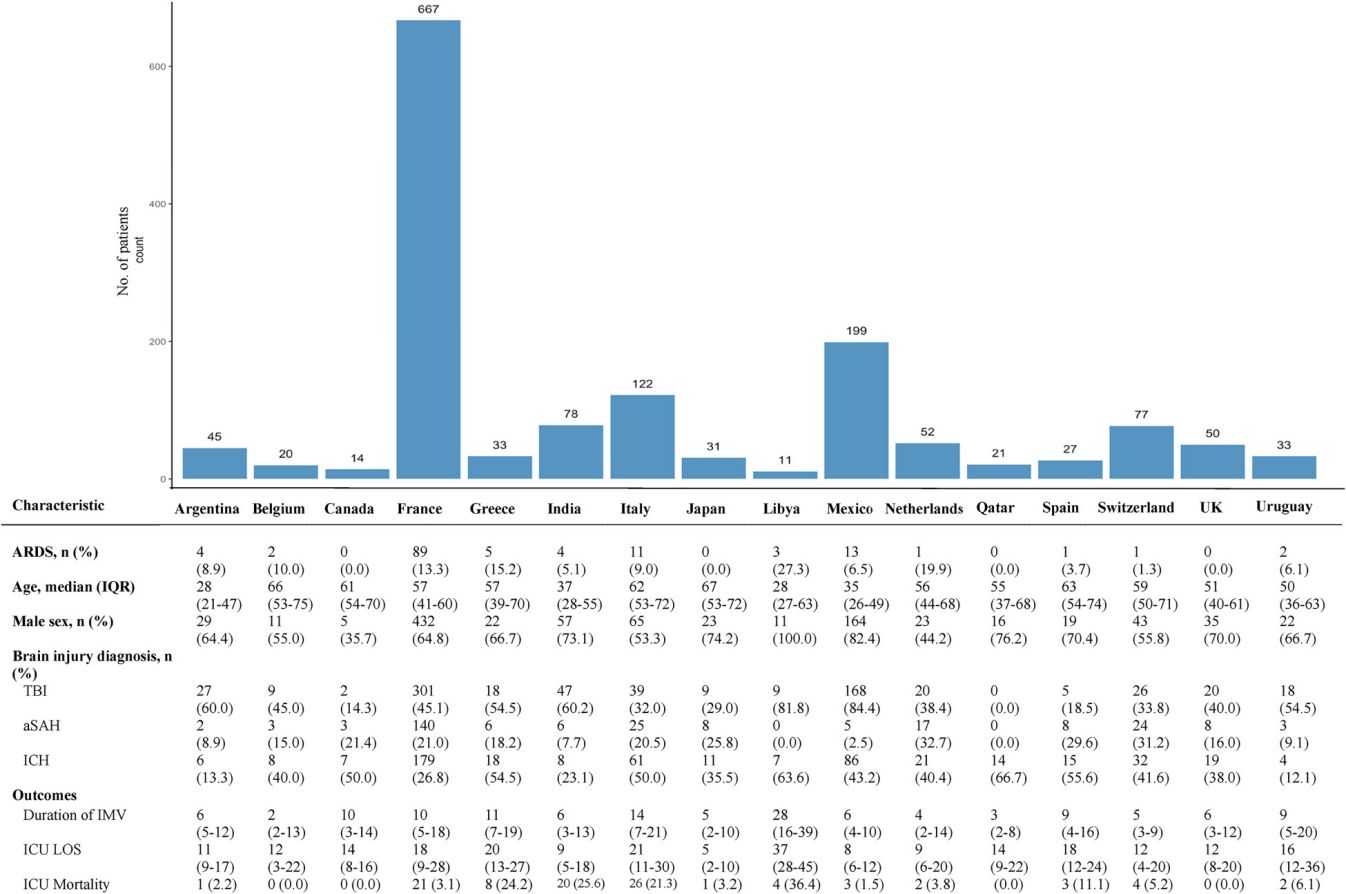
Baseline characteristics by country.

Abbreviations: ARDS, acute respiratory distress syndrome; aSAH, aneurysmal subarachnoid hemorrhage; ICH, intracranial hemorrhage; IMV, invasive mechanical ventilation; IQR, interquartile range; ICU, intensive care unit; LOS, length of stay TBI, traumatic brain injury; UK, United Kingdom.

From 1512 patients in the primary study, we included 1492 patients (98.7%) with non-missing ARDS status from 67 hospital sites and 16 countries (Figures 1 and 2). Table 1, Figure 2, and eFigure 2 show baseline characteristics of the included patients stratified by ARDS status, country of admission, and world income region, respectively. Baseline characteristics of patients excluded due to missing ARDS status are reported in eTable 1. The median age of the overall cohort was 54 years (IQR 36-66), 986 patients (66.1%) were male, and the median Glasgow Coma Scale score before intubation was 7 (IQR 5-9). The most common admission diagnosis was TBI (720 patients; 48.2% of the cohort). Most patients were recruited from Europe (1048 patients; 70.2% of the cohort). Patients from high-income countries comprised 77.5% of the study sample, were older, and had lower admission Glasgow Coma Scale scores than those of patients from upper-middle income countries or low-middle income countries (eFigure 2).

**Table 1.** Baseline Characteristics Stratified by ARDS Status.

Characteristic	Overall, n = 1,492^a^	No ARDS, n = 1355	ARDS, n = 137	*P*-value^b^
Age, median (IQR)	54 (36-66)	54 (36-67)	54 (32-63)	.08
Male sex, n (%)	986 (66.1)	878 (64.8)	108 (78.8)	**<** **.** **001**
Pre-intubation GCS, median (IQR)	7 (5-9)	7 (5-9)	7 (5-8)	.88
Comorbidities, n (%)				
Hypertension	442 (29.6)	407 (30.0)	35 (25.5)	.27
Diabetes	181 (12.1)	164 (12.1)	17 (12.4)	.92
Congestive heart failure	44 (3.0)	39 (2.9)	5 (3.6)	.59
Pulmonary disease	50 (3.4)	43 (3.2)	7 (5.1)	.22
Malignancy	68 (4.6)	64 (4.7)	4 (2.9)	.33
Active tobacco use	326 (22.0)	284 (21.0)	42 (30.6)	.**01**
Brain injury diagnosis^c^, n (%)				
TBI	720 (48.2)	636 (46.9)	84 (61.3)	.**001**
aSAH	263 (17.6)	230 (17.0)	33 (24.1)	.**04**
ICH	511 (34.2)	483 (35.6)	28 (20.4)	**<**.**001**
AIS	141 (9.5)	135 (10.0)	6 (4.4)	.**03**
CNS infection	73 (4.9)	70 (5.2)	3 (2.2)	.12
Brain tumor	72 (4.8)	68 (5.0)	4 (2.9)	.28
Day 1 ventilatory variables, median (IQR)				
Tidal volume, mL/kg PBW	7.1 (6.5-7.9)	7.2 (6.5-8.0)	6.8 (6.2-7.4)	**<**.**001**
Respiratory rate	16 (14-19)	16 (14-18)	18 (16-22)	**<**.**001**
Plateau pressure, cm H_2_O	16 (14-19)	16 (14-19)	17 (14-20)	.**01**
PEEP, cm H_2_O	5 (5-6)	5 (5-6)	6 (5-7)	**<**.**001**
Driving pressure, cm H_2_O	10 (8-13)	10 (8-13)	11 (9-14)	.**03**
PaO_2_/FiO_2_	310 (230-417)	314 (237-422)	280 (179-374)	**<**.**001**
ICU events, n (%)				
Decompressive craniectomy	287 (19.2)	251 (18.5)	36 (26.3)	.**03**
ICP probe	693 (46.4)	549 (40.5)	84 (61.3)	**<**.**001**
Therapeutic hypothermia	61 (4.1)	48 (3.5)	13 (9.5)	.**001**
Barbiturate coma	83 (5.6)	70 (5.2)	13 (9.5)	.**04**
VAP	594 (39.8)	476 (35.1)	118 (86.1)	**<**.**001**
Outcomes				
Duration of IMV, median (IQR)	8 (4-16)	8 (4-14)	20 (12-28)	**<**.**001**
ICU LOS, median (IQR)	14 (8-24)	13 (7-23)	27 (19-39)	**<**.**001**
Tracheostomy, n (%)	410 (27.6)	366 (27.2)	44 (32.6)	
Extubation failure, n (%)	250 (21.4)	212 (19.9)	38 (36.9)	**<**.**001**
ICU mortality, n (%)	95 (6.4)	77 (5.8)	18 (13.1)	**<**.**001**

Abbreviations: AIS, acute ischemic stroke; ARDS, acute respiratory distress syndrome; aSAH; aneurysmal subarachnoid hemorrhage; CNS, central nervous system; GCS, Glasgow Coma Scale; ICH, intracranial hemorrhage; ICP, intracranial pressure; ICU, intensive care unit; IMV, invasive mechanical ventilation; IQR, interquartile range; LOS, length of stay; PaO_2_/FiO_2_, ratio of partial pressure of oxygen to fraction of inspired oxygen concentration; PBW, predicted body weight; PEEP, positive end expiratory pressure; TBI, traumatic brain injury; VAP, ventilator-associated pneumonia.

^a^ 1492 of 1512 patients from the original study had non-missing ARDS status.

^b^ *P* values are for the comparison between patients with no ARDS (*n* = 1355) versus those with ARDS (*n* = 137).

^c^ Some patients had more than one type of brain injury recorded on admission, such that column totals exceed the number of patients per group. Bold values indicate statistically significant differences.

### Incidence of ARDS

ARDS occurred in 137 patients with ABI (9.2% of overall cohort). ARDS was seen in 18/95 patients who died in ICU (18.9%) and 23/168 patients who died during their index hospitalization (13.7%). Across countries, as shown in Figure 2, the median ARDS incidence was 5.1% (IQR 0.0-10.0; range 0-27.3); variation in ARDS incidence across countries was not significant after adjustment for patient-level covariates (eFigure 3). Across world income regions, ARDS incidence was 9.8% in high-income countries, 7.8% in upper-middle income countries, and 4.9% in lower-middle income countries (eFigure 2).

### Ventilation and Sedation Characteristics

Ventilation and sedation data on days 1, 3, and 7 of IMV, stratified by ARDS status at any point during ICU admission, are reported in Table 2. On each day of IMV, patients with ARDS had a higher respiratory rate, higher positive end expiratory pressure, higher driving pressure, higher plateau pressure, and lower tidal volume (except for IMV day 1) than those of patients without ARDS. More frequently, patients with ARDS received midazolam and neuromuscular blockers on days 1, 3, and 7 of IMV. The use of propofol was higher on days 3 and 7 in patients with ARDS; dexmedetomidine use was similar between patients with and without ARDS on each day of IMV. Adherence to lung-protective ventilation targets (defined as tidal volume ≤8 mL/kg predicted body weight, plateau pressure ≤30 cm H_2_O, and driving pressure ≤15 cm H_2_O) in patients with ARDS on each day of IMV was high, as reported in eTable 2.

**Table 2.** Ventilation and Sedation Variables Stratified by ARDS Status^a^.

Variable	Day 1			Day 3			Day 7		
	No ARDS	ARDS	*P* value	No ARDS	ARDS	*P* value	No ARDS	ARDS	*P* value
Ventilation									
Tidal volume, mL/kg PBW, median (IQR)	6.5 (7.2-8.0)	6.8 (6.2-7.4)	**<**.**001**	7.2 (6.5-8.1)	6.7 (6.2-7.6)	**<**.**001**	7.4 (6.6-8.4)	6.9 (6.3-7.7)	**<**.**001**
Respiratory rate, median (IQR)	16 (14-18)	18 (16-22)	**<**.**001**	16 (14-20)	20 (16-22)	**<**.**001**	18 (15-22)	22 (18-26)	**<**.**001**
Plateau pressure, cm H_2_O, median (IQR)	16 (14-19)	17 (14-20)	.**009**	16 (14-19)	18 (15-22)	**<**.**001**	16 (13-20)	20 (16-24)	**<**.**001**
PEEP, cm H_2_O, median (IQR)	5 (5-6)	6 (5-7)	**<**.**001**	5 (5-7)	6 (5-8)	**<**.**001**	6 (5-8)	8 (6-10)	**<**.**001**
Driving pressure, cm H_2_O, median (IQR)	10 (8-13)	11 (9-14)	.**034**	10 (8-13)	11 (9-14)	.**008**	10 (7-13)	12 (8-15)	.**001**
Sedation and NMBA									
Any sedation	1183 (87.3)	126 (91.2)	.113	756 (55.8)	118 (86.2)	**<**.**001**	352 (30.0)	98 (71.5)	**<**.**001**
Propofol	813 (60.3)	90 (65.7)	.219	494 (48.7)	85 (66.4)	**<**.**001**	210 (30.4)	65 (55.6)	**<**.**001**
Midazolam	614 (45.5)	81 (59.1)	**<**.**001**	386 (37.9)	82 (64.1)	**<**.**001**	139 (20.1)	67 (57.3)	**<**.**001**
Dexmedetomidine	30 (2.2)	3 (2.2)	.977	66 (6.5)	6 (4.7)	.422	81 (11.7)	7 (6.0)	.067
NMBA, n (%)	69 (5.1)	16 (11.7)	.**002**	40 (4.0)	29 (22.6)	**<**.**001**	26 (3.8)	38 (32.5)	**<**.**001**

Abbreviations: ARDS, acute respiratory distress syndrome; IQR, interquartile range; NMBA, neuromuscular blocking agents; PBW, predicted body weight; PEEP, positive end expiratory pressure.

^a^ Time-varying ventilatory data are compared for patients with and without ARDS on days 1, 3, and 7 of invasive mechanical ventilation. Comparisons are reported for patients who developed ARDS at any timepoint during the index ICU admission (ie, ARDS could have occurred before or after the indicated days). Bold values indicate statistically significant differences.

### Outcomes

*ARDS and ICU Mortality*. In unadjusted analysis, development of ARDS was associated with increased ICU mortality (OR, 2.50; 95% CI, 1.41-4.23). In the mixed-effect analysis adjusted for patient-level factors and country of admission, the association between ARDS and increased ICU mortality persisted (OR 2.45; 95% CI, 1.19-5.06). There was no association between increasing severity of ARDS and ICU mortality, but effect estimates were imprecise (eTable 3). The *E*-value for the association between ARDS and ICU mortality was 4.33 (lower bound of 95% CI, 3.50), indicating that an unmeasured confounder would need to have a relative risk of at least 4.33 to overcome the observed association between ARDS and ICU mortality.

**Figure 3.**
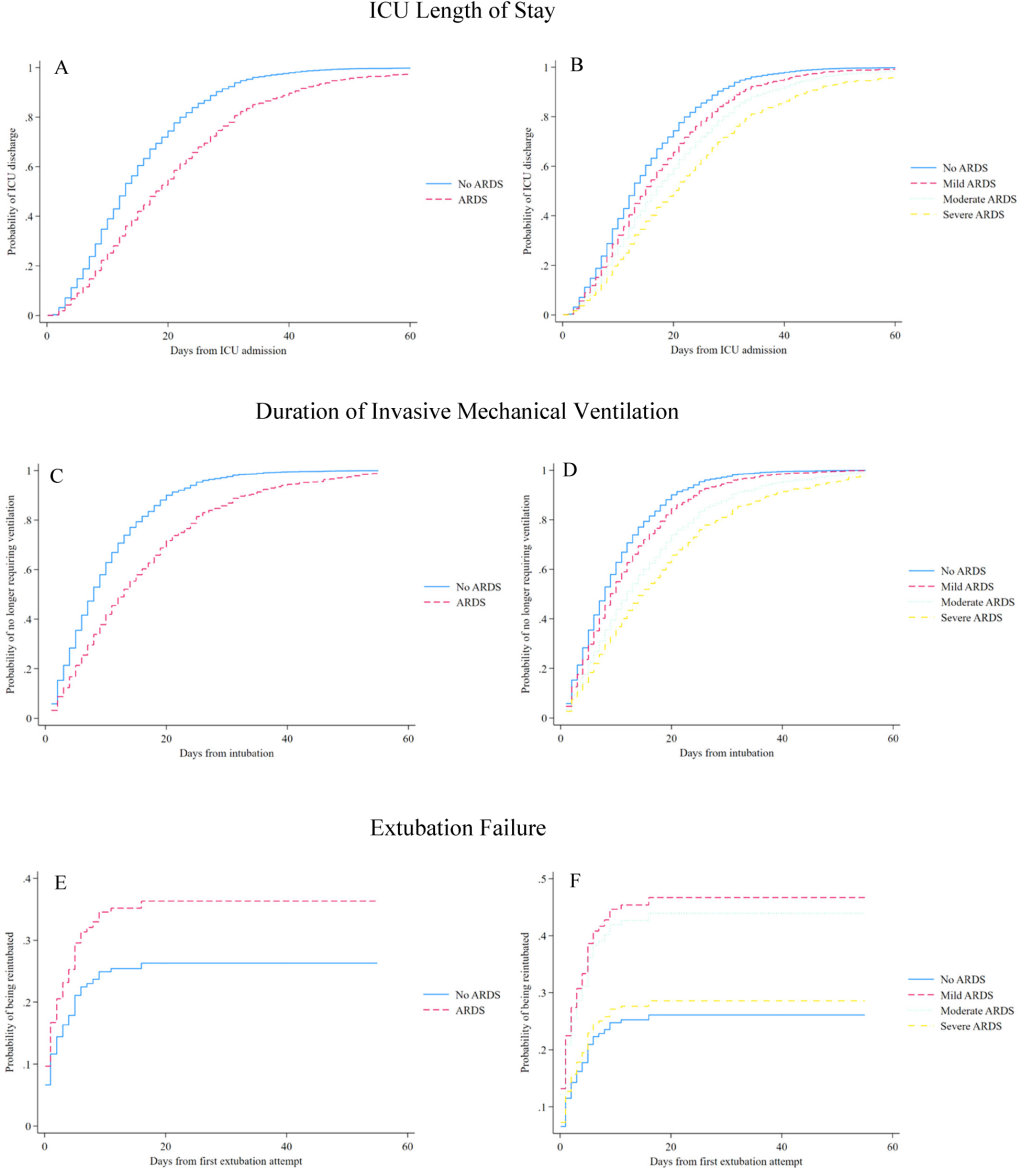
ARDS and additional outcomes. Adjusted time-to-event analyses for additional outcomes are shown. Models A, C, and E are reported for patients with ARDS versus those without ARDS. Models B, D, and F are reported with ARDS according to severity, using the worse PaO_2_/FiO_2_ recorded during ICU admission. Hazard ratios <1 for the models of ICU length of stay and duration of invasive mechanical ventilation indicate longer time to each event.

*ARDS and Additional Outcomes*. Results of the analysis for additional outcomes are reported in Figure 3. ARDS was independently associated with longer ICU LOS (adjusted HR 0.59; 95% CI, 0.48-0.73) and longer duration of IMV (adjusted HR 0.54; 95% CI, 0.44-0.67). In the analysis stratified by ARDS severity, moderate and severe (but not mild) ARDS were associated with longer ICU LOS and longer duration of IMV, and all associations became progressively stronger with increasing ARDS severity (Figure 3 and eTable 3). The *E*-values for models evaluating ICU LOS and duration of IMV were 2.06 (lower limit of 95% CI, 1.27) and 2.43 (lower limit of 95% CI, 1.44), respectively. ARDS was not associated with extubation failure (HR 1.48; 95% CI 0.99-2.21).

*Subgroup Analysis*. The results of the subgroup analysis are presented in eTable 4. The association between ARDS and excess ICU mortality persisted in patients with TBI, aSAH, and ICH as their primary diagnosis, but not in patients with other admission diagnoses.

## Discussion

In this preplanned secondary analysis of the ENIO study, including 1492 patients with ABI from 67 hospital sites and 16 countries, the incidence of ARDS in individuals surviving beyond liberation from IMV was 9.2%. Median ARDS incidence across countries was 5.1%, and development of ARDS was independently associated with higher ICU mortality, longer ICU LOS, and longer duration of IMV. Adherence to lung-protective ventilation targets in the overall cohort, and specifically among patients with ARDS, was high. These data highlight the incidence, management, and clinical impact of ARDS in patients with ABI and suggest the importance of remaining vigilant to patients recovering from ARDS in the ICU, even when illness severity appears ostensibly reduced following liberation from IMV.

ARDS is an important mediator of additional neurologic injury and worse clinical outcomes in patients with ABI. Proposed mechanisms include immune system dysregulation, brain tissue hypoxia resulting from systemic hypoxemia, and release of pro-inflammatory cytokines with subsequent blood-brain barrier disruption and penetration into the central nervous system.^11,30,31^ Ventilatory strategies adopted in response to ARDS may impose additional risk of neurologic injury; for example, via low tidal volume ventilation leading to hypercapnia and elevated intracranial pressure (ICP) or higher positive end expiratory pressure leading to alveolar overdistension, elevated ICP, and decreased cerebral perfusion pressure.^13,32^ Although our study was not designed to investigate mechanistic pathways, it does suggest their aggregate downstream impact on clinical outcomes in a diverse cohort of patients with ABI. This information could support future translational research to clarify the pathophysiologic processes underlying our described associations.

We found that variation in ARDS incidence in brain-injured patients across countries was not significant after adjustment for patient factors. This result should be viewed as exploratory in light of our small sample size and low proportion of patients with ARDS. The LUNG SAFE study, published in 2016, included 29 144 critically ill patients from 459 ICUs worldwide and reported important differences in ARDS incidence across regions, with a higher incidence in Europe and North America compared to Asia and Africa.^10^ Although the difference in results is likely explained by the increased statistical power of LUNG SAFE, it remains possible that regional variation has become less prominent over time due to clinical factors, for example, due to increased international uptake of established criteria for ARDS diagnosis,^22^ improvement in clinician recognition of ARDS,^33^ increasing awareness of the negative impacts of a missed ARDS diagnosis,^34^ and higher adoption of lung-protective ventilation strategies with time.^14^ The high compliance with multiple lung-protective targets observed among patients with ARDS in this sub-study supports the latter hypothesis.

Of note, the incidence of ARDS in this study (9.2%) is lower than that reported in other cohorts of patients with ABI.^9,16,35^ This could relate to the mixed composition of ENIO, where the inclusion of a large number of patients with a low baseline risk of ARDS (eg, patients with brain tumors, intracranial infection, and acute ischemic stroke) could have reduced the cohort-level incidence of ARDS. Indeed, this subset accounted for 286 patients (19.2% of the sub-study cohort) yet only 13/137 cases of ARDS (9.5%). The low ARDS incidence could also be explained by early and robust compliance with lung-protective ventilatory strategies such that downstream ARDS risk was attenuated,^36^ missed cases of true ARDS due to clinician under-recognition,^34^ and exclusion of patients who died before liberation from IMV in the original ENIO study.^20^ In addition, the use of the Berlin definition for exposure ascertainment could have led to non-classification of ARDS in clinical settings where arterial blood gas analysis and chest radiography were not routinely available.^37^

The observational nature of this study precludes a causal interpretation of its results. However, the association between ARDS and worse clinical outcomes has high face validity and is consistent with existing findings, including in patients with and without ABI.^6,19,38,39^ For some outcomes in this analysis, the associations followed an expected gradient, in which effect sizes strengthened as ARDS severity worsened. Findings from our sensitivity analysis suggested that residual confounding was unlikely to negate our study's conclusions. For example, the *E*-value for the association between ARDS and ICU mortality was 4.33. The odds ratio from the same model for ventilator-associated pneumonia (VAP), a robust and well-established confounder, was 1.46. It is unlikely there exists an unmeasured confounder with a threefold higher effect size than that of VAP on the association between ARDS and ICU mortality.

Strengths of this study include its large sample size and inclusion of patients with diverse etiologies of brain injury. The existing literature is largely informed by single-center studies.^16,18,40,41^ ENIO was conducted across 73 ICUs worldwide,^20^ and findings of this sub-study are generalizable to multiple practice locations and resource settings. This study also has limitations. First, without chest radiographs or additional clinical context, we were unable to verify site-submitted cases of ARDS, such that true cases may have been missed and non-cases may have been classified as true cases. This misclassification was likely non-differential across outcome categories and independent of other errors, such that our study findings would be conservatively biased towards the null.^42,43^ Second our results could be susceptible to residual confounding related to the absence of recorded information on variables known to be associated with ARDS in the primary study.^20^ As indicated in our sensitivity analysis, the existence of a confounder with the effect size required to overcome the exposure-outcome relationship is clinically unlikely. Nevertheless, it remains possible that ARDS is simply an epiphenomenon of increased illness severity and not the causal driver of worse outcomes. Third, the association between ARDS and clinical outcomes has been shown to vary with the severity of hypoxemic respiratory failure.^10,44,45^ Our subgroup analysis for ICU mortality did not replicate this finding, but effect estimates were imprecise as reflected by wide confidence intervals, and results should be interpreted with caution. Fourth, our results could be influenced by systematic differences in patients with missing ARDS status who were excluded from the present study. However, baseline characteristics of these patients were comparable to included patients, providing indirect evidence of similarity between groups and arguing against major biases arising due to patient selection. Finally, our results could be susceptible to immortal time bias. We were unable to account for immortal time, since the day of ARDS development was unknown. However, since the majority of ARDS cases after ABI occur early (in patients with TBI and aSAH, median onset is 3 days following injury),^46,47^ the number of patients with ARDS developing late in their ventilatory course is expected to be small and therefore unlikely to substantially modify results.

## Conclusion

In this analysis of 1492 brain-injured patients from 16 countries and 67 ICUs surviving beyond liberation from IMV, the incidence of ARDS was 9.2%. Development of ARDS during ICU admission was associated with increased ICU mortality, increased ICU LOS, and longer duration of IMV. Future studies are needed to elucidate potential mechanisms related to our described associations.

## Supplemental Material

sj-pdf-1-jic-10.1177_08850666231194532 - Supplemental material for Incidence and Outcomes of Acute Respiratory Distress Syndrome in Brain-Injured Patients Receiving Invasive Ventilation: A Secondary Analysis of the ENIO Study Click here for additional data file. Supplemental material, sj-pdf-1-jic-10.1177_08850666231194532 for Incidence and Outcomes of Acute Respiratory Distress Syndrome in Brain-Injured Patients Receiving Invasive Ventilation: A Secondary Analysis of the ENIO Study by Shaurya Taran, Robert D. Stevens, Bastien Perrot, Victoria A. McCredie, Raphael Cinotti, Karim Asehnoune, Paolo Pelosi, Chiara Robba and in Journal of Intensive Care Medicine
