# Supplementary material for: Incidence and Outcomes of Acute Respiratory Distress Syndrome in Brain-Injured Patients Receiving Invasive Ventilation: A Secondary Analysis of the ENIO Study
Source: J Intensive Care Med. 2023 Aug 11;39(2):136–45. doi: 10.1177/08850666231194532 (PMC10771027; doi:10.1177/08850666231194532)
Supplement: sj-pdf-1-jic-10.1177_08850666231194532 - Supplemental material for Incidence and Outcomes of Acute Respiratory Distress Syndrome in Brain-Injured Patients Receiving Invasive Ventilation: A Secondary Analysis of the ENIO Study [file sj-pdf-1-jic-10.1177_08850666231194532.pdf]

Incidence and Outcomes of Acute Respiratory Distress Syndrome in Brain-Injured Patients Receiving Invasive Ventilation: A Secondary Analysis of the ENIO Study

Shaurya Taran, Robert D. Stevens, Bastien Perrot, Victoria A. McCredie, Raphael Cinotti, Karim Asehnoune, Paolo Pelosi, Chiara Robba

Online Supplement

**eFigure 1:** Directed acyclic graph modeling putative exposure-outcome relationship	2
**eAppendix 1:** Additional methods	3
**eAppendix 2:** Sample codes	7
**eFigure 2:** Baseline characteristics by world income region	8
**eTable 1:** Baseline characteristics of patients with missing ARDS status	9
**eTable 2:** Compliance with evidence-based ventilatory thresholds	10
**eFigure 3:** Unadjusted and adjusted variation in ARDS incidence by country	11
**eTable 3:** Outcomes according to ARDS severity	12
**eTable 4:** ARDS and ICU mortality according to admission diagnosis	13

eFigure 1: Directed acyclic graph modeling putative exposure-outcome relationship

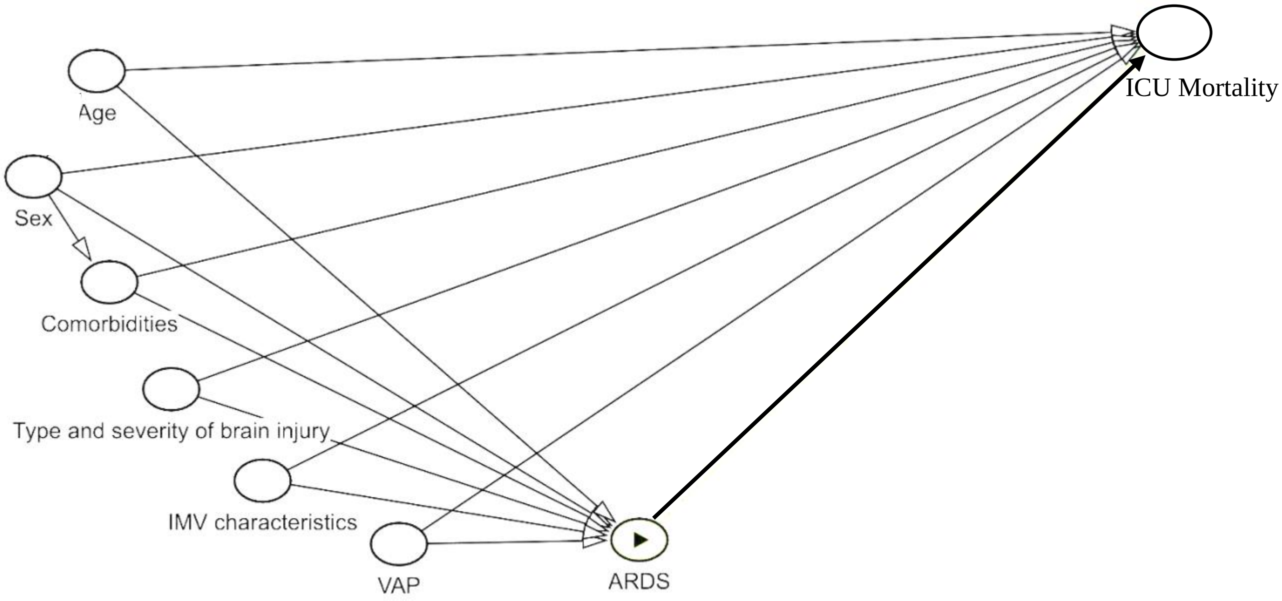

Putative relationships between the primary exposure, confounders, and primary outcome are modeled in the above directed acyclic graph. Comorbidities is a vector that includes congestive heart failure and pre-existing pulmonary disease. Type and severity of brain injury is a vector that includes the neurologic admission diagnosis, presence of intracranial monitor, requirement for decompressive craniectomy, and lowest Glasgow Coma Score before intubation. IMV characteristics included plateau pressure, positive end expiratory pressure (PEEP), driving pressure, and respiratory rate. Only variables recorded on the first day of mechanical ventilation were included to maximize the probability that characteristics preceded the exposure. Ventilator-associated pneumonia was selected based on its established association between the primary exposure and primary outcome. To prevent overfitting of the model, not all of the covariates indicated here were included. Selection

Abbreviations: ABI, Acute brain injury; ARDS, acute respiratory distress syndrome; ICU, Intensive care unit; IMV, invasive mechanical ventilation; LOS length of stay; VAP, ventilator associated pneumonia

Figure created with www.daggity.net

eAppendix 1: Additional methods

ARDS diagnosis

ARDS occurrence (yes/no) was recorded by site reviewers according to the Berlin definition [1]. Patients were identified as meeting the primary exposure definition if they fulfilled the Berlin criteria at any point during the index ICU admission. Patients were further classified as having mild, moderate, or severe ARDS according to the worst PaO_2/FiO_2 ratio after meeting Berlin definition criteria. All models for clinical outcomes were fitted with ARDS as the primary exposure. Twenty patients (1.3% of the original study population) had missing information on ARDS occurrence and were excluded from the present secondary analysis.

ARDS Incidence

We reported the incidence of ARDS in patients surviving beyond liberation from invasive mechanical ventilation (IMV) in i) the overall cohort, ii) within individual countries, and iii) within world income regions. We used the 2022 World Bank Countries classification to classify countries as high-income countries (HIC), upper-middle income countries (UMIC), and low-middle income countries (LMIC). Low-income countries were not represented in the original study [2]. In an exploratory analysis, we examined whether ARDS incidence varied significantly across countries. We reported both the unadjusted and adjusted variation in ARDS incidence (the latter using fixed-effect patient covariates).

Clinical outcomes

The primary clinical outcome was ICU mortality. Additional outcomes were ICU length of stay (LOS), duration of IMV, and extubation failure. We defined extubation failure as the unplanned need for reintubation at any point during the index ICU admission following the index liberation attempt. In the absence of a universal definition for extubation failure, we selected a longer timeframe to maximize the use of available data from the ENIO study. Some patients had signs and symptoms of respiratory failure in the post-extubation setting and received “rescue” use of non-invasive respiratory support with high flow nasal cannula (HFNC) or non-invasive positive pressure ventilation (NIPPV). We did not classify the need for rescue modalities as extubation failure (only patients in whom invasive ventilation was reinstated were considered to meet the outcome definition).

Patients with a primary tracheostomy (i.e., tracheostomy with no prior extubation attempt) were excluded from the analysis of extubation failure, since they were ineligible to experience the outcome of interest. For all other analyses, we retained tracheostomized patients.

Covariate selection and model development

Additional covariates for inclusion in statistical models were selected using a subject matter approach and further narrowed based on percent missingness in the dataset [3]. Associations were modeled using a directed acyclic graph (DAG) to ensure appropriate covariate adjustment [4]. Data-driven selection procedures (e.g., stepwise methods, statistical significance in univariable analysis, change in effect estimates) were not used to identify or refine the pool of eligible covariates. We conditioned on known or putative confounders identified a-priori, as reported in our DAG. Following these procedures, we adjusted the ICU mortality model for age, sex, traumatic brain injury, lowest Glasgow Coma Score on ICU admission, presence of intracranial

pressure probe (used as a marker to indicate potential elevated intracranial pressure), decompressive craniectomy, driving pressure on day 1 of invasive ventilation, and ventilator-associated pneumonia. Models for ICU length of stay and duration of IMV were adjusted using age, sex, brain injury diagnosis, and ventilator-associated pneumonia. The model for extubation failure was adjusted using age, sex, lowest Glasgow Coma Score on extubation day, type of extubation (accidental vs planned), time to first extubation attempt (in days), and airway protective factors (cough and swallow).

All clinical outcomes were evaluated using mixed effect models. ICU mortality (the primary outcome) was evaluated using a logistic mixed model with country as the random intercept. ICU length of stay, duration of IMV, and extubation failure (additional outcomes) were evaluated using mixed effect Cox proportional hazards models with a shared frailty term for patients managed in the same country. This modeling structure reflects expected similarities in clinical management among patients managed in the same region. Standard model diagnostics were performed to evaluate modeling assumptions. Multicollinearity was evaluated using the variance inflation factor (VIF). In case multicollinearity was identified, the covariate with a higher *a-priori* relevance in the causal structure (based on existing literature, biological plausibility, or expert opinion) was selected for inclusion in the final model.

Additional analyses

We examined the association between ARDS and ICU mortality within subgroups of patients according to their neurologic diagnosis at ICU admission. We used a minimal set of adjustment covariates to prevent overfitting within these smaller patient groups. Subgroup samples were not mutually exclusive (i.e., patients in the original study could have more than

one neurologic diagnosis recorded at ICU admission) [2]. We retained these overlapping subgroup assignments in the present secondary analysis.

In a sensitivity analysis, we calculated E-values for all models to quantify the impact of unmeasured confounders on our described associations [5]. The E-value describes the minimum strength of association that an unmeasured confounder should have with both the exposure and outcome to explain away the observed association, conditional on measured confounders [5,6]. Larger E-values suggest that a greater degree of unmeasured confounding is necessary to negate the association. All E-values were reported along with the lower bound of the 95% confidence interval, following current recommendations [6,7].

We used STATA v.17.0 and the R statistical packages *lme4* and *ggplot2* to develop the models and plots reported in this analysis.

eAppendix 2: Sample codes

Sample codes are provided to illustrate the modeling process.

Model for ICU mortality (testing random intercept)

```
M1<-glmer(ICU_MORTALITY~ 1 + (1|COUNTRY),
      data = E,
      control = glmerControl(optimizer = "bobyqa"),
      family=binomial(link="logit"))

summary(M1)

M2<-glm(ICU_MORTALITY~1, data=E, family=binomial)

summary(M2)

anova(M1, M2, test="Chisq") # Model with random intercept fits significantly better, with lower AIC
```

Model for ICU mortality (full mixed model with ARDS as primary exposure)

```
M3<-glmer(ICU_MORTALITY~ ARDS_ICU + GENDER + PATIENT_AGE + TRAUMTIC_BRAIN_INJURY +
LOWEST_GLASGOW_INCLUSION + D1_DeltaP + NOSOCOMIAL_VAP_ICU +
DECOMPRESSIVE_CRANIECTOMY + INTRA_CRANIAL_PROBE + 1 + (1|COUNTRY),
       data = E,
       control = glmerControl(optimizer = "bobyqa"),
       family=binomial(link="logit"))

summary(M3)

M4<-glm(ICU_MORTALITY~ ARDS_ICU + GENDER + PATIENT_AGE + TRAUMTIC_BRAIN_INJURY +
LOWEST_GLASGOW_INCLUSION + D1_DeltaP + NOSOCOMIAL_VAP_ICU +
DECOMPRESSIVE_CRANIECTOMY + INTRA_CRANIAL_PROBE,
          data = E, family=binomial) # Fixed-effect only model

summary(M4)

anova(M3, M4, test="Chisq") # Model with random intercept fits significantly better, with lower AIC
```

eFigure 2: Baseline characteristics by world income region

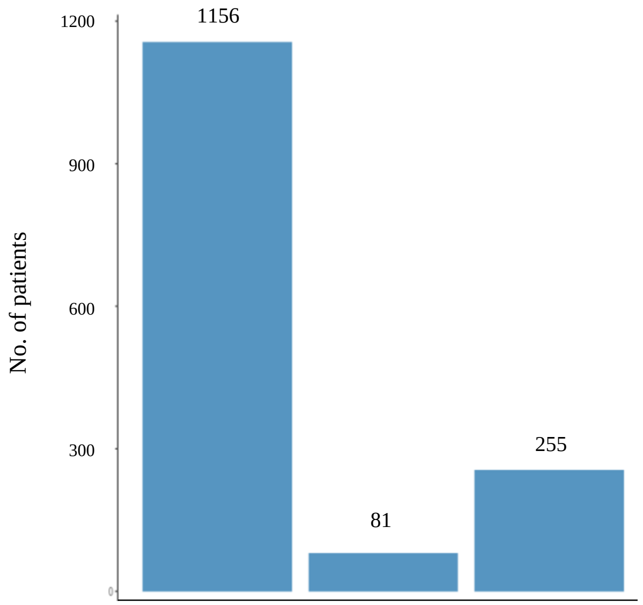

Characteristics	HIC	LMIC	UMIC	P-value
**ARDS, n (%)**	113 (9.8%)	20 (7.8%)	4 (4.9%)	0.20
**Age, median (IQR)**	58 (45-68)	37 (28-54)	34 (25-50)	**<0.001**
**Male sex, n (%)**	723 (62.5%)	59 (72.8%)	204 (80.0%)	**<0.001**
**GCS, median (IQR)**	7 (4-9)	7 (5-9)	7 (6-9)	**0.006**
**Brain injury diagnosis, n (%)** [a]				
TBI	467 (40.4)	49 (60.5)	204 (80.0)	**<0.001**
aSAH	248 (21.4)	6 (7.4)	9 (3.5)	**<0.001**
ICH	392 (33.9)	20 (24.7)	99 (38.8)	0.057
**Outcomes**				
Duration of IMV	9 (4-18)	6 (3-12)	6 (4-11)	**<0.001**
ICU LOS	16 (9-27)	9 (5-16)	9 (6-13)	**<0.001**
ICU Mortality	67 (5.8%)	20 (27.0%)	8 (3.1%)	**<0.001**

[a] Some patients had more than one type of brain injury recorded on admission, such that column totals exceed the number of patients per group
Abbreviations: ARDS, acute respiratory distress syndrome; aSAH, aneurysmal subarachnoid hemorrhage; GCS, Glasgow coma score; HIC: high income country; ICH, intracranial hemorrhage; IMV, invasive mechanical ventilation; IQR, interquartile range; ICU, intensive care unit; LMIC, low-middle income country; TBI, traumatic brain injury; UK, United Kingdom; UMIC, upper-middle income country

eTable 1: Baseline characteristics of patients with missing ARDS status

Characteristic	ARDS status known, n = 1,492 [a]	ARDS status unknown, n = 20 [b]	P-value
Age, median (IQR)	54 (36-66)	60 (52-67)	0.20
Male sex, n (%)	986 (66.1)	12 (60.0)	0.60
Pre-intubation GCS, median (IQR)	7 (5-9)	7 (5-10)	0.50
Comorbidities, n (%)			
Hypertension	442 (29.6)	9 (45.0)	0.14
Diabetes	181 (12.1)	2 (10.0)	0.90
Congestive heart failure	44 (3.0)	0 (0.0)	0.90
Pulmonary disease	50 (3.4)	1 (5.0)	0.50
Malignancy	68 (4.6)	0 (0.0)	0.90
Active tobacco use	326 (22.0)	4 (21.0)	0.90
Brain injury diagnosis [b], n (%)			
TBI	720 (48.2)	6 (30.0)	0.10
aSAH	263 (17.6)	6 (30.0)	0.15
ICH	511 (34.2)	12 (60)	**0.02**
AIS	141 (9.5)	0 (0.0)	0.20
CNS infection	73 (4.9)	1 (5.0)	0.90
Brain tumor	72 (4.8)	0 (0.0)	0.60
Day 1 ventilatory variables, median (IQR)			
Tidal volume, ml/kg PBW	7.1 (6.5-7.9)	7.5 (6.9-7.9)	0.30
Respiratory rate	16 (14-19)	15 (12-16)	**0.01**
Plateau pressure, cm H_2O	16 (14-19)	17 (14-21)	0.20
PEEP, cm H_2O	5 (5-6)	6 (5-6)	0.40
Driving pressure, cm H_2O	10 (8-13)	11.5 (8-15.5)	0.40
PaO_2/FiO_2	310 (230-417)	244 (178-352)	0.14
ICU events, n (%)			
Decompressive craniectomy	287 (19.2)	4 (21.0)	0.80
ICP probe	693 (46.4)	9 (47.4)	0.70
Therapeutic hypothermia	61 (4.1)	0 (0.0)	0.90
Barbiturate coma	83 (5.6)	3 (15.8)	0.09

[a] Column composed of patients that were included in the sub-study
[b] Column composed of patients that were not included in the sub-study
[b] Some patients had more than one type of brain injury recorded on admission, such that column totals exceed the number of patients per group
Abbreviations: AIS, acute ischemic stroke; ARDS, acute respiratory distress syndrome; aSAH; aneurysmal subarachnoid hemorrhage; CNS, central nervous system; GCS, Glasgow Coma Scale; ICH, intracranial hemorrhage; ICP, intracranial pressure; ICU, intensive care unit; IQR, interquartile range; PaO_2/FiO_2, ratio or partial pressure of oxygen to fraction of inspired oxygen concentration; PBW, predicted body weight; PEEP, positive end expiratory pressure; SD, standard deviation; TBI, traumatic brain injury; VAP, ventilator associated pneumonia

eTable 2: Compliance with evidence-based ventilatory thresholds [a]

Variable	% Compliance		
	Day 1	Day 3	Day 7
Tidal volume ≤ 8 mL/kg PBW	84.7	78.1	73.0
Plateau pressure ≤ 30 cm H_2O	84.7	80.3	70.1
Driving pressure ≤ 15 cm H_2O	74.4	72.3	58.4

[a] The proportion of patients receiving ventilatory care compliant with evidence-based thresholds for ARDS on days 1, 3, and 7 of invasive mechanical ventilation are reported. Ventilatory thresholds are derived from international best-practice guidelines. Proportions in the table are indicated for patients who developed ARDS at any time point during the index ICU admission (i.e., ARDS could have occurred before or after the indicated days).
Abbreviations: PBW, predicted body weight

eFigure 3: Unadjusted and adjusted variation in ARDS incidence by country [a]

A: Random intercepts from unadjusted model

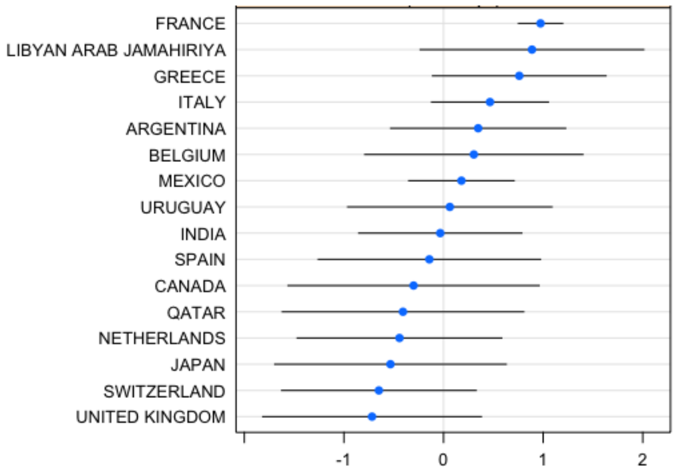

B: Random intercepts from adjusted model

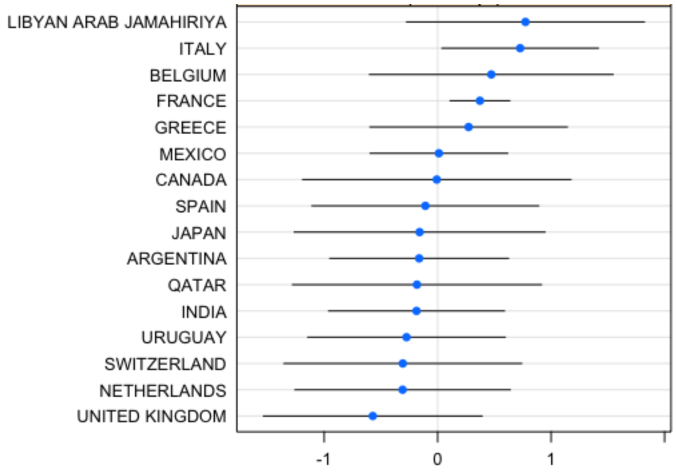

[a] In the unadjusted analysis (panel A), ARDS incidence varied significantly by country (random intercept-only model, compared to null model, $\chi 2 = 21.362$, $p<0.001$). However, after adjusting for patient-level covariates (panel B), the variation in ARDS incidence across countries was no longer significant ($\chi 2 = 1.666$, $p=0.198$).

eTable 3: Outcomes according to ARDS severity

Outcome	OR/HR [a]	95% CI	P-value
**ICU Mortality**			
Mild	1.95	0.36-10.53	0.44
Moderate	2.58	0.91-7.34	0.07
Severe	2.58	0.96-6.93	0.06
**ICU Length of Stay**			
Mild	0.80	0.51-1.26	0.33
Moderate	0.66	0.49-0.90	**0.008**
Severe	0.52	0.38-0.69	**<0.001**
**Duration of IMV**			
Mild	0.82	0.52-1.30	0.40
Moderate	0.57	0.42-0.78	**<0.001**
Severe	0.46	0.34-0.61	**<0.001**
**Extubation Failure**			
Mild	2.08	0.90-4.79	0.08
Moderate	1.91	1.07-3.41	**0.028**
Severe	1.11	0.64-1.93	0.38

[a] OR is for the model of ICU mortality. HR is for all other outcomes.
The ICU mortality model was adjusted for age, sex, traumatic brain injury, lowest Glasgow Coma Score on ICU admission, presence of intracranial pressure probe (used as a marker to indicate potential elevated intracranial pressure), decompressive craniectomy, driving pressure on day 1 of invasive ventilation, and ventilator-associated pneumonia. Models for ICU length of stay and duration of IMV were adjusted using age, sex, brain injury diagnosis, and ventilator-associated pneumonia. The model for extubation failure was adjusted using age, sex, lowest Glasgow Coma Score on extubation day, type of extubation (accidental vs planned), time to first extubation attempt (in days), and airway protective factors (cough and swallow). In all models, ARDS was included as a categorical variable (mild vs moderate vs severe). Associations are represented against a reference level of "no ARDS." Associations < 1 for the models of ICU length of stay and duration of IMV represent a longer time in ICU and longer time receiving IMV, respectively.
Abbreviations: ARDS, acute respiratory distress syndrome; CI, confidence interval; HR, hazard ratio; ICU, intensive care unit; IMV, invasive mechanical ventilation; OR, odds ratio

eTable 4: ARDS and ICU mortality according to admission diagnosis

Diagnosis [a]	OR	95% CI
TBI	6.79	2.20-20.85
aSAH	4.08	1.55-14.44
ICH	4.14	1.37-12.48
Other	0.30	0.01-3.42

[a] The subgroup model was adjusted using a minimal set of covariates to prevent overfitting. Associations are reported for ARDS of any severity, against a reference level of no ARDS, within subgroups defined by brain injury diagnosis on admission.
Abbreviations: ARDS, acute respiratory distress syndrome; aSAH; aneurysmal subarachnoid hemorrhage; CI, confidence interval; ICU, intensive care unit; IMV, mechanical ventilation, invasive mechanical ventilation; ICH, intracranial hemorrhage; IRR, incident rate ratio; LOS, length of stay; OR, odds ratio; TBI, traumatic brain injury

References for online supplement

1. Ranieri VM, Rubenfeld GD, Thompson BT, et al. Acute respiratory distress syndrome: the Berlin Definition. Jama 2012;307:2526-33.

2. Cinotti R, Mijangos JC, Pelosi P, et al. Extubation in neurocritical care patients: the ENIO international prospective study. Intensive Care Med 2022;48:1539-50.

3. VanderWeele TJ. Principles of confounder selection. Eur J Epidemiol 2019;34:211-9.

4. Lipsky AM, Greenland S. Causal Directed Acyclic Graphs. JAMA 2022;327:1083-4.

5. VanderWeele TJ, Ding P. Sensitivity Analysis in Observational Research: Introducing the E-Value. Ann Intern Med 2017;167:268-74.

6. Haneuse S, VanderWeele TJ, Arterburn D. Using the E-Value to Assess the Potential Effect of Unmeasured Confounding in Observational Studies. JAMA 2019;321:602-3.

7. VanderWeele TJ, Mathur MB. Commentary: Developing best-practice guidelines for the reporting of E-values. Int J Epidemiol 2020;49:1495-7.
